# *KCNJ11* and *KCNQ1* Gene Polymorphisms and Placental Expression in Women with Gestational Diabetes Mellitus

**DOI:** 10.3390/genes13081315

**Published:** 2022-07-23

**Authors:** Sandra Majcher, Przemysław Ustianowski, Damian Malinowski, Michał Czerewaty, Maciej Tarnowski, Krzysztof Safranow, Violetta Dziedziejko, Andrzej Pawlik

**Affiliations:** 1 Department of Physiology, Pomeranian Medical University, 70-111 Szczecin, Poland; majchersk@gmail.com (S.M.); michal.czerewaty@wp.pl (M.C.); maciejt@pum.edu.pl (M.T.); 2 Department of Nursing, Pomeranian Medical University, 70-111 Szczecin, Poland; przemyslaw.ustianowski@pum.edu.pl; 3 Department of Experimental and Clinical Pharmacology, Pomeranian Medical University, 70-111 Szczecin, Poland; damian.malinowski@pum.edu.pl; 4 Department of Biochemistry and Medical Chemistry, Pomeranian Medical University, 70-111 Szczecin, Poland; chrissaf@mp.pl (K.S.); viola@pum.edu.pl (V.D.)

**Keywords:** KCNJ11, KCNQ1, polymorphism, GDM

## Abstract

Gestational diabetes mellitus (GDM) represents carbohydrate intolerance in pregnant women. The pathogenesis of GDM is very complex, but abnormalities in insulin production and secretion underlie the disease. Potassium channels play an important role in insulin production and secretion. The family of potassium channels includes (among others) the potassium inwardly rectifying channel, subfamily J, member 11 (KCNJ11) and voltage-gated K+ channel (*KCNQ1*). The aim of the study was to examine the distribution of the *KCNJ11* rs5219 and *KCNQ1* rs151290 and rs2237892 gene polymorphisms in women with GDM and pregnant women with normal carbohydrate tolerance, to verify whether these polymorphisms are risk factors for GDM. This study included 204 Caucasian pregnant women with GDM and 207 pregnant women with normal glucose tolerance (NGT) from the West Pomeranian region of Poland. The diagnosis of GDM was based on a 75 g oral glucose tolerance test (OGTT) at 24–28 weeks gestation. There were no statistically significant differences in distribution of the *KCNJ11* rs5219 and *KCNQ1* rs151290 and rs2237892 gene polymorphisms between women with GDM and pregnant women with normal carbohydrate tolerance. Moreover, there were no statistically significant associations between the studied genotypes and the selected clinical parameters in women with GDM. The results of our study suggest that the *KCNJ11* rs5219 and *KCNQ1* rs2237892 and rs151290 gene polymorphisms are not significant risk factors associated with the development of GDM in our population. There were also no differences in the expression of *KCNJ11* and *KCNQ1* genes in the placenta of women with GDM and normal carbohydrate tolerance. However, an association between *KCNJ11* gene expression in placenta and APGAR score in newborns was found.

## 1. Introduction

Gestational diabetes mellitus (GDM) is carbohydrate intolerance occurring in pregnant women. GDM may lead to various metabolic complications; therefore, factors predisposing to the development of GDM are being searched out [1]. The pathogenesis of GDM is complex and includes risk factors, such as age, obesity and family history of diabetes [2]. Previous studies have shown that some genetic loci predisposing to the development of type 2 diabetes mellitus may also predispose to GDM [3]. In GDM, impaired insulin secretion has been shown [4,5]. Potassium channels are involved in the synthesis and secretion of insulin [6]. The family of potassium channels includes (among others) the potassium inwardly rectifying channel, subfamily J, member 11 (KCNJ11) and voltage-gated K+ channel (*KCNQ1*). The *KCNJ11* gene is located 4.5 Kb from *ABCC8* on chromosome 11p15.1 and has a single exon encoding for the 390 amino acid Kir6.2 protein, whereas the *KCNQ1* gene is located on 11p15.5, encoding the pore-forming a-subunit of the voltage-gated K+ channel (KvLQT1) [7,8]. The expression of potassium channels has been detected in various cells and tissues, including pancreatic islet cells. In pancreatic beta cells, these channels play an important role in insulin synthesis and secretion [9]. In the *KCNJ11* and *KCNQ1* genes, several single nucleotide polymorphisms have been detected, which may alter mRNA and protein expression (rs5219, rs2237892 and rs151290). These polymorphisms have been associated with type 2 diabetes mellitus in various populations [10,11,12]. The aim of the study was to examine the distribution of the *KCNJ11* rs5219 and *KCNQ1* rs151290 and rs2237892 gene polymorphisms in women with GDM and pregnant women with normal carbohydrate tolerance, to verify whether these polymorphisms are risk factors for GDM. In addition, we examined the association between these polymorphisms and selected clinical parameters to check if the studied polymorphisms had an influence on them. We also examined the expression of *KCNJ11* and *KCNQ1* genes in women with GDM and normal carbohydrate tolerance. We conducted the research in a group of Caucasian women from the West Pomeranian region of Poland.

## 2. Materials and Methods

### 2.1. Participants

This study included 204 Caucasian pregnant women with GDM and 207 pregnant women with normal glucose tolerance (NGT) from the West Pomeranian region of Poland. The diagnosis of GDM was based on a 75 g oral glucose tolerance test (OGTT) at 24–28 weeks gestation, according to the International Association of Diabetes and Pregnancy Study Groups (IADPSG) criteria [13]. The diagnosis of GDM was made when one of the following plasma glucose values in the OGTT was met or exceeded: fasting plasma glucose 92 mg/dL (5.1 mmol/L), 1 h plasma glucose 180 mg/dL (10.0 mmol/L) or 2 h plasma glucose 153 mg/dL (8.5 mmol/L). Among the pregnant women with GDM, 78% of them were treated with diet control alone throughout their pregnancies, while the remaining 22% of them were treated with diet control and insulin until delivery. All pregnant women were without any acute or chronic complications, such as diabetic ketoacidosis, or other disorders affecting glucose metabolism. The study was approved by the Ethics Committee of Pomeranian Medical University, Szczecin, Poland, and written informed consent was obtained from all subjects.

### 2.2. Methods

All samples were genotyped in duplicate using allelic discrimination assays with TaqMan^®^ probes (Applied Biosystems, Carlsbad, CA, USA) on a 7500 Fast Real-Time PCR Detection System (Applied Biosystems). In order to discriminate the polymorphisms, we employed TaqMan^®^ Pre-Designed SNP Genotyping Assays, including appropriate primers and fluorescently labelled (FAM and VIC) MGB™ probes to detect the alleles.

### 2.3. Determination of KCNJ11 and KCNQ1 Gene Expression in Placenta

#### 2.3.1. RNA Isolation

For this study, placentas were obtained from randomly selected 39 women with GDM and 29 healthy women who had a natural delivery after 37 weeks of gestation. All samples were collected at the Department of Obstetrics and Gynecology, Pomeranian Medical University in Szczecin. After delivery whole placenta was placed in 0.9% NaCl and immediately transported to the Department of Physiology. The placental samples of approximately 100 mg were then excised from the maternal side of the cotyledons for RNA extraction. No visible connective tissue, vessels and calcium deposits were detected. Total RNA was extracted from homogenates using the RNeasy Fibrous Tissue Mini Kit (Qiagen, Hilden, Germany) in accordance with the manufacturer’s protocol. The concentration and purity of RNA samples was determined by measuring the absorbance using a spectrophotometer Perkin Elmer Lambda Bio+ (PerkinElmer Waltham, MA, USA).

#### 2.3.2. Real-Time Quantitative Reverse-Transcription PCR (RQ-PCR)

An amount of 0.4 μg of isolated mRNA from each sample was reverse transcribed into cDNA in a total volume of 20 μL using a cDNA synthesis kit (RevertAid RT Kit, Thermo Scientific, Waltham, MA, USA) according to the manufacturer’s protocol. The analysis of the quantitative expression of *KCNJ11* and *KCN*

*BMG shouQ1* genes as well as the reference gene, was performed using real-time RT-PCR on an ABI PRISM^®^ Fast 7500 Sequence Detection System (Applied Biosystems) as previously described [14]. To normalize mRNA levels between different samples, we used β-2 microglobulin (*BMG*) as reference gene. The reference gene was determined based on the available literature [15,16,17]. Each sample was analyzed in two technical replications, and mean cycle threshold (CT) values were used for further analysis. Each reaction (20 μL) contained 2 μL of cDNA dilution. To calculate the values, the 2^−^^ΔCt^ method was used.

### 2.4. Statistical Analysis

The consistency of the genotype distribution with Hardy–Weinberg equilibrium (HWE) was assessed using the exact test. A chi-square (χ^2^) test was used to compare the genotype and allele distributions between the groups. Quantitative variables were compared between the genotype groups using the Mann–Whitney test. A multivariate logistic regression model was used to find independent predictors of GDM risk. *p*-values < 0.05 were considered statistically significant.

## 3. Results

The distributions of the studied polymorphisms were in the HWE (*p* > 0.05). The distribution of studied polymorphisms in women with GDM and control women is shown in Table 1. As shown in Table 1, there are no statistically significant differences in distribution of the *KCNJ11* rs5219 and *KCNQ1* rs151290 and rs2237892 gene polymorphisms between women with GDM and pregnant women with normal carbohydrate tolerance.

We also compared the distribution of the *KCNJ11* rs5219 and *KCNQ1* rs151290 and rs2237892 gene polymorphisms between women with GDM treated with insulin and pregnant women with normal carbohydrate tolerance, women with GDM treated with diet control and pregnant women with normal carbohydrate tolerance, as well as women with GDM treated with insulin with women with GDM treated with diet control. As shown in Table 2, Table 3 and Table 4, there are no statistically significant differences in the distributions of the studied polymorphisms between these groups.

We also examined the associations between the *KCNJ11* rs5219 and *KCNQ1* rs151290 and rs2237892 gene polymorphisms and clinical parameters, such as body mass before pregnancy, body mass at birth, body mass increase during pregnancy, BMI before pregnancy, BMI at birth, BMI increase during pregnancy, glycated haemoglobin (HbA1c), daily insulin requirement, child birth time, newborn body mass and APGAR score in women with GDM (Table 5, Table 6 and Table 7). The majority of associations between the above parameters and the *KCNJ11* rs5219 and *KCNQ1* rs151290 and rs2237892 genotypes was statistically insignificant. We only observed lower BMI and HbA1c values in women with the *KCNQ1* rs2237892 TT genotype in comparison with women with the CC and TT genotypes. Additionally, the newborns from women with the CC genotype had higher APGAR scores in comparison with the newborns from women with the CT genotype.

Additionally, we examined the expression of *KCNJ11* and *KCNQ1* genes in the placenta of women with and without GDM. *KCNJ11* expression in the placenta of women with GDM and controls was 0.012 ± 0.057 and 0.013 ± 0.054, respectively. These differences were statistically non-significant (*p* = 0.95). *KCNQ1* expression in the placenta of women with GDM and controls was 0.0044 ± 0.0035 and 0.0043 ± 0.0039, respectively. These differences were statistically non-significant (*p* = 0.72).

We also examined correlations between expression in the placenta of *KCNJ11* and *KCNQ1* genes in women with GDM and clinical parameters. There were no statistically significant correlations between *KCNQ1* gene expression in the placenta and clinical parameters. Expression of the *KCNJ11* gene correlated significantly with the APGAR score in newborns (Table 8 and Table 9).

## 4. Discussion

In this study, we examined the distribution of the *KCNJ11* rs5219 and *KCNQ1* rs2237892 and rs151290 gene polymorphisms in women with GDM and pregnant women with normal carbohydrate tolerance, to verify whether these polymorphisms were risk factors for GDM and might influence the selected clinical parameters of women with GDM and their newborns. Our results suggest a lack of statistically significant associations between these polymorphisms and the risk of GDM in our population from the West Pomeranian region of Poland. The association of the *KCNQ1* rs2237892 genotypes with some clinical parameters was difficult to interpret due to the fact that only two women were carriers of the TT genotype. This relationship will have to be confirmed in a much larger population of women with GDM.

There were also no statistically significant differences between *KCNJ11* and *KCNQ1* gene expression in placenta of women with and without GDM. There were no statistically significant correlations between *KCNQ1* gene expression in the placenta and clinical parameters. Expression of the *KCNJ11* gene correlated significantly with the APGAR score in newborns.

Potassium channels are expressed in many tissues, including pancreatic islet cells, and are involved in insulin production and secretion and, thus, in carbohydrate metabolism and the pathogenesis of type 2 diabetes mellitus and GDM [5,6]. Numerous studies have examined the polymorphisms in the *KCNJ11* and *KCNQ1* genes as the risk factors for type 2 diabetes mellitus; nevertheless, the results of the studies are inconsistent and are different in various populations [10,11,12]. Several studies conducted mainly in Asian populations examined the associations between the *KCNQ1* gene polymorphisms and GDM. The results of these studies were inconsistent. In a study carried out in a Chinese population, the rs2237892 C allele was associated with an increased risk of GDM and higher glucose levels [18]. Additionally, in studies by Shin et al. and Kwak et al., the *KCNQ1* rs2237892 gene polymorphism was associated with GDM in Korean women [19,20]. The meta-analysis by Mao et al. indicated that the *KCNQ1* rs2237892 C allele might be associated with the increased risk of GDM [21]. The *KCNQ1* rs2237892 gene polymorphism was also associated with GDM risk in Mexican women [22]. Stuebe et al. have shown that the *KCNQ1* rs2237892 gene polymorphism is associated with gestational weight gain in American women [23]. In the studies by Wang et al. and by Chon et al., there were no statistically significant associations between the KCNQ1 gene polymorphisms and GDM in Chinese and Korean women [24,25].

The results of studies examining the association between the *KCNJ11* rs5219 gene polymorphism and GDM are also inconsistent. The meta-analyses by Zhang et al. and Mao et al. indicated that the *KCNJ11* rs5219 T allele might be a risk factor for GDM [21,26]. In the study by Cho et al., there were no statistically significant associations between the *KCNJ11* rs5219 gene polymorphism and GDM in Korean population [27]. This association was also not confirmed in Greek and Swedish women [28,29].

The observed variations between these studies may be mainly due to ethnic differences and the influence of other environmental factors that increase the risk of GDM. Potassium channels play an important role in the synthesis and secretion of insulin and in glucose metabolism. Previous studies have shown impaired function of potassium channels in patients with diabetes [5,6]. The *KCNJ11* and *KCNQ1* gene polymorphisms were associated with type 2 diabetes mellitus and GDM [10,11,12]. The pathogenesis of GDM is very complex. It is based on complex disorders of insulin secretion and insulin resistance. So far, the influence of many genetic factors, including genetic polymorphisms, on the pathogenesis of GDM has been demonstrated. These factors may increase the risk of developing GDM and may also influence some clinical parameters of the disease. New genetic factors that could increase the risk of developing this disease are constantly being searched for.

## 5. Conclusions

The results of our study suggest that the *KCNJ11* rs5219 and *KCNQ1* rs2237892 and rs151290 gene polymorphisms are not significant risk factors associated with the development of GDM in women from the West Pomeranian region of Poland. There were also no differences in the expression of *KCNJ11* and *KCNQ1* genes in the placenta of women with GDM and normal carbohydrate tolerance.

However, an association between *KCNJ11* gene expression in placenta and APGAR score in newborns was found. Nevertheless, the understanding of the exact role of potassium channels and the polymorphisms of their genes in the pathogenesis of GDM requires further studies on large groups of patients.

## Figures and Tables

**Table 1:** Distribution of *KCNJ11* and *KCNQ1* genotypes in GDM women and control group.

	Control Group		GDM		*p* Value ^^^		*p* Value ^^^	OR (95% CI)
	n	%	n	%				
***KCNJ11*** **rs5219** **genotype**								
CC	83	40.10%	79	38.73%	0.52	TT + CT vs. CC	0.78	1.06 (0.71–1.57)
CT	93	44.93%	101	49.51%		TT vs. CT + CC	0.34	0.76 (0.43–1.34)
TT	31	14.98%	24	11.76%		TT vs. CC	0.51	0.81 (0.44–1.51)
						CT vs. CC	0.54	1.14 (0.75–1.73)
						TT vs. CT	0.27	0.71 (0.39–1.30)
**Allele**								
C	259	62.56%	259	63.48%		T vs. C	0.78	0.96 (0.72–1.28)
T	155	37.44%	149	36.52%				
***KCNQ1*** **rs151290** **genotype**								
CC	117	56.52%	113	55.39%	0.88	AA + CA vs. CC	0.82	1.05 (0.71–1.55)
CA	76	36.71%	79	38.73%		AA vs. CA + CC	0.71	0.86 (0.39–1.91)
AA	14	6.76%	12	5.88%		AA vs. CC	0.77	0.89 (0.39–2.00)
						CA vs. CC	0.72	1.08 (0.72–1.62)
						AA vs. CA	0.65	0.82 (0.36–1.90)
**Allele**								
C	310	74.88%	305	74.75%		A vs. C	0.97	1.01 (0.73–1.38)
A	104	25.12%	103	25.25%				
***KCNQ1*** **rs2237892** **genotype**								
CC	181	87.44%	172	84.31%	0.65	TT + CT vs. CC	0.36	1.30 (0.74–2.26)
CT	24	11.59%	30	14.71%		TT vs. CT + CC	0.99	1.01 (0.14–7.27)
TT	2	0.97%	2	0.98%		TT vs. CC	0.96	1.05 (0.15–7.55)
						CT vs. CC	0.35	1.32 (0.74–2.34)
						TT vs. CT	0.83	0.80 (0.10–6.10)
**Allele**								
C	386	93.24%	374	91.67%		T vs. C	0.39	1.25 (0.75–2.11)
T	28	6.76%	34	8.33%				

^ χ^2^ test; HWE: control group *p* = 0.55, GDM group *p* = 0.37 for *KCNJ11* rs5219. HWE: control group *p* = 0.71, GDM group *p* = 0.85 for *KCNQ1* rs151290. HWE: control group *p* = 0.23, GDM group *p* = 0.63 for *KCNQ1* rs2237892.

**Table 2:** Distribution of *KCNJ11* and *KCNQ1* genotypes in GDM women treated with insulin and control group.

	Control Group		GDM Women Treated with Insulin		*p* Value ^^^		*p* Value ^^^	OR (95% CI)
	n	%	n	%				
***KCNJ11*** **rs5219** **genotype**								
CC	83	40.10%	21	40.38%	0.96	TT + CT vs. CC	0.97	0.99 (0.53–1.84)
CT	93	44.93%	24	46.15%		TT vs. CT + CC	0.78	0.88 (0.37–2.14)
TT	31	14.98%	7	13.46%		TT vs. CC	0.81	0.89 (0.35–2.31)
						CT vs. CC	0.95	1.02 (0.53–1.97)
						TT vs. CT	0.78	0.88 (0.34–2.23)
**Allele**								
C	259	62.56%	66	63.46%		T vs. C	0.87	0.96 (0.62–1.50)
T	155	37.44%	38	36.54%				
***KCNQ1*** **rs151290** **genotype**								
CC	117	56.52%	27	51.92%	0.75	AA + CA vs. CC	0.55	1.20 (0.65–2.21)
CA	76	36.71%	22	42.31%		AA vs. CA + CC	0.80	0.84 (0.23–3.05)
AA	14	6.76%	3	5.77%		AA vs. CC	0.91	0.93 (0.25–3.46)
						CA vs. CC	0.48	1.25 (0.67–2.36)
						AA vs. CA	0.66	0.74 (0.19–2.81)
**Allele**								
C	310	74.88%	76	73.08%		A vs. C	0.71	1.10 (0.67–1.79)
A	104	25.12%	28	26.92%				
***KCNQ1*** **rs2237892** **genotype**								
CC	181	87.44%	43	82.69%	0.43	TT + CT vs. CC	0.37	1.46 (0.64–3.33)
CT	24	11.59%	9	17.31%		CT vs. CC	0.28	1.58 (0.68–3.64)
TT	2	0.97%	0	0.00%				
**Allele**								
C	386	93.24%	95	91.35%		T vs. C	0.50	1.31 (0.60–2.86)
T	28	6.76%	9	8.65%				

^ χ^2^ test.

**Table 3:** Distribution of *KCNJ11* and *KCNQ1* genotypes in GDM women on diet and control group.

	Control Group		GDM Women on Diet		*p* Value ^^^		*p* Value ^^^	OR (95% CI)
	n	%	n	%				
***KCNJ11*** **rs5219** **genotype**								
CC	83	40.10%	58	38.16%	0.44	TT + CT vs. CC	0.71	1.08 (0.71–1.67)
CT	93	44.93%	77	50.66%		TT vs. CT + CC	0.30	0.71 (0.38–1.35)
TT	31	14.98%	17	11.18%		TT vs. CC	0.48	0.78 (0.40–1.55)
						CT vs. CC	0.46	1.18 (0.75–1.86)
						TT vs. CT	0.22	0.66 (0.34–1.29)
**Allele**								
C	259	62.56%	193	63.49%		T vs. C	0.80	0.96 (0.71–1.31)
T	155	37.44%	111	36.51%				
***KCNQ1*** **rs151290** **genotype**								
CC	117	56.52%	86	56.58%	0.95	AA + CA vs. CC	0.99	1.00 (0.65–1.52)
CA	76	36.71%	57	37.50%		AA vs. CA + CC	0.75	0.87 (0.37–2.06)
AA	14	6.76%	9	5.92%		AA vs. CC	0.77	0.87 (0.36–2.11)
						CA vs. CC	0.93	1.02 (0.66–1.59)
						AA vs. CA	0.74	0.86 (0.35–2.12)
**Allele**								
C	310	74.88%	229	75.33%		A vs. C	0.89	0.98 (0.69–1.38)
A	104	25.12%	75	24.67%				
***KCNQ1*** **rs2237892** **genotype**								
CC	181	87.44%	129	84.87%	0.78	TT + CT vs. CC	0.48	1.24 (0.68–2.27)
CT	24	11.59%	21	13.82%		TT vs. CT + CC	0.76	1.37 (0.19–9.81)
TT	2	0.97%	2	1.32%		TT vs. CC	0.74	1.40 (0.20–10.09)
						CT vs. CC	0.52	1.23 (0.66–2.30)
						TT vs. CT	0.90	1.14 (0.15–8.84)
**Allele**								
C	386	93.24%	279	91.78%		T vs. C	0.46	1.24 (0.70–2.16)
T	28	6.76%	25	8.22%				

^ χ^2^ test.

**Table 4:** Distribution of *KCNJ11* and *KCNQ1* genotypes in GDM women treated with insulin and GDM women on diet.

	GDM Women on Diet		GDM Women Treated with Insulin		*p* Value ^^^		*p* Value ^^^	OR (95% CI)
	n	%	n	%				
***KCNJ11*** **rs5219** **genotype**								
CC	58	38.16%	21	40.38%	0.83	TT + CT vs. CC	0.78	0.91 (0.48–1.73)
CT	77	50.66%	24	46.15%		TT vs. CT + CC	0.66	1.24 (0.48–3.17)
TT	17	11.18%	7	13.46%		TT vs. CC	0.80	1.14 (0.41–3.13)
						CT vs. CC	0.66	0.86 (0.44–1.70)
						TT vs. CT	0.58	1.32 (0.49–3.56)
**Allele**								
C	193	63.49%	66	63.46%		T vs. C	1.00	1.00 (0.63–1.59)
T	111	36.51%	38	36.54%				
***KCNQ1*** **rs151290** **genotype**								
CC	86	56.58%	27	51.92%	0.83	AA+ CA vs. CC	0.56	1.21 (0.64–2.27)
CA	57	37.50%	22	42.31%		AA vs. CA +CC	0.97	0.97 (0.25–3.74)
AA	9	5.92%	3	5.77%		AA vs. CC	0.93	1.06 (0.27–4.20)
						CA vs. CC	0.54	1.23 (0.64–2.37)
						AA vs. CA	0.84	0.86 (0.21–3.49)
**Allele**								
C	229	75.33%	76	73.08%		A vs. C	0.65	1.12 (0.68–1.87)
A	75	24.67%	28	26.92%				
***KCNQ1*** **rs2237892** **genotype**								
CC	129	84.87%	43	82.69%	0.60	TT + CT vs. CC	0.71	1.17 (0.50–2.73)
CT	21	13.82%	9	17.31%		CT vs. CC	0.56	1.29 (0.55–3.02)
TT	2	1.32%	0	0.00%				
**Allele**								
C	279	91.78%	95	91.35%		T vs. C	0.89	1.06 (0.48–2.35)
T	25	8.22%	9	8.65%				

^ χ^2^ test.

**Table 5:** Clinical parameters of women with GDM stratified according to *KCNJ11* rs5219 genotype.

Parameters	*KCNJ11* rs5219 Genotype					
	CC n = 79	CT n = 101	TT n = 24	CC vs. CT	CC vs. TT	CT vs. TT
	Mean ± SD	Mean ± SD	Mean ± SD	*p* ^&^		
Body mass before pregnancy [kg]	66.6 ± 13.8	69.1 ± 17.9	70.8 ± 17.8	0.61	0.43	0.58
Body mass at birth [kg]	78.2 ± 14.9	80.0 ± 18.3	81.2 ± 19.0	0.87	0.73	0.86
Body mass increase during pregnancy [kg]	11.6 ± 5.7	10.9 ± 4.9	10.4 ± 5.0	0.54	0.29	0.57
BMI before pregnancy [kg/m^2^]	24.8 ± 4.7	25.3 ± 6.1	25.5 ± 6.0	0.80	0.99	0.62
BMI at birth [kg/m^2^]	29.2 ± 5.3	29.3 ± 6.2	29.2 ± 6.3	0.62	0.74	0.99
BMI increase during pregnancy [kg/m^2^]	4.4 ± 2.2	4.0 ± 1.8	3.7 ± 1.8	0.45	0.17	0.44
HbA1c [%]	5.48 ± 0.50	5.61 ± 0.46	5.59 ± 0.43	0.03	0.26	0.75
Daily insulin requirement [unit]	5.10 ± 11.16	5.36 ± 11.26	5.54 ± 13.57	0.83	0.86	0.75
Childbirth [weeks pregnant]	38.7 ± 1.7	38.3 ± 2.2	38.8 ± 0.9	0.25	0.68	0.69
Newborn body mass [g]	3380 ± 515	3176 ± 688	3265 ± 686	0.04	0.17	0.98
APGAR [0–10 points]	9.7 ± 0.9	9.7 ± 1.0	9.6 ± 1.1	0.32	0.59	0.92

^&^ U Mann–Whitney test.

**Table 6:** Clinical parameters of women with GDM stratified according to *KCNQ1* rs151290 genotype.

Parameters	*KCNQ1* rs151290 Genotype					
	CC n = 113	CA n = 79	AA n = 12	CC vs. CA	CC vs. AA	CA vs. AA
	Mean ± SD	Mean ± SD	Mean ± SD	*p* ^&^		
Body mass before pregnancy [kg]	69.5 ± 17.9	67.7 ± 14.7	62.0 ± 9.8	0.71	0.17	0.23
Body mass at birth [kg]	80.6 ± 18.5	78.9 ± 15.9	73.0 ± 9.2	0.81	0.22	0.23
Body mass increase during pregnancy [kg]	11.1 ± 5.2	11.2 ± 5.4	11.0 ± 5.4	0.99	0.97	0.95
BMI before pregnancy [kg/m^2^]	25.4 ± 5.9	24.9 ± 5.3	23.7 ± 3.8	0.63	0.48	0.59
BMI at birth [kg/m^2^]	29.5 ± 6.0	29.1 ± 5.9	28.0 ± 4.0	0.72	0.62	0.75
BMI increase during pregnancy [kg/m^2^]	4.1 ± 1.9	4.2 ± 2.1	4.3 ± 2.2	0.93	0.83	0.95
HbA1c [%]	5.57 ± 0.47	5.55 ± 0.46	5.58 ± 0.63	0.70	0.59	0.61
Daily insulin requirement [unit]	5.23 ± 12.08	4.97 ± 10.05	7.75 ± 14.55	0.62	0.74	0.93
Childbirth [weeks pregnant]	38.3 ± 2.1	38.7 ± 1.6	38.3 ± 1.4	0.03	0.37	0.14
Newborn body mass [g]	3252 ± 673	3306 ± 590	3125 ± 475	0.68	0.27	0.19
APGAR [0–10 points]	9.7 ± 1.1	9.7 ± 0.9	9.8 ± 0.6	0.73	0.61	0.76

^&^ U Mann–Whitney test.

**Table 7:** Clinical parameters of women with GDM stratified according to *KCNQ1* rs2237892 genotype.

Parameters	*KCNQ1* rs2237892 Genotype					
	CC n = 172	CT n = 30	TT n = 2	CC vs. CT	CC vs. TT	CT vs. TT
	Mean ± SD	Mean ± SD	Mean ± SD	*p* ^&^		
Body mass before pregnancy [kg]	68.6 ± 16.9	68.0 ± 12.8	50.0 ± 7.1	0.73	0.05	0.03
Body mass at birth [kg]	79.8 ± 17.5	78.7 ± 14.7	59.0 ± 11.3	0.86	0.06	0.07
Body mass increase during pregnancy [kg]	11.2 ± 5.4	10.7 ± 4.0	9.0 ± 4.2	0.80	0.56	0.61
BMI before pregnancy [kg/m^2^]	25.2 ± 5.7	24.9 ± 4.5	18.6 ± 2.3	0.78	0.04	0.02
BMI at birth [kg/m^2^]	29.4 ± 6.0	28.8 ± 5.1	21.9 ± 3.8	0.74	0.04	0.05
BMI increase during pregnancy [kg/m^2^]	4.2 ± 2.1	3.9 ± 1.5	3.3 ± 1.5	0.71	0.54	0.64
HbA1c [%]	5.57 ± 0.47	5.57 ± 0.50	4.79 ± 0.13	0.94	0.02	0.02
Daily insulin requirement [unit]	5.16 ± 11.44	6.30 ± 11.97	0.00 ± 0.00	0.54	0.42	0.38
Childbirth [weeks pregnant]	38.5 ± 1.9	38.2 ± 2.1	37.0 ± 2.8	0.70	0.30	0.42
Newborn body mass [g]	3271 ± 615	3268 ± 728	2725 ± 233	0.49	0.09	0.16
APGAR [0–10]	9.8 ± 0.8	9.2 ± 1.6	10.0 ± 0.0	0.005	0.65	0.41

^&^ U Mann–Whitney test.

**Table 8:** Correlations between *KCNJ11* expression in the placenta and clinical parameters in the GDM group.

Parameters Correlated with Placental Expression of *KCNJ11*	Rs	*p*
Age [years]	−0.13	0.55
Fasting glucose [mg/dl]	0.11	0.61
Daily insulin requirement [unit]	−0.08	0.72
Body mass before pregnancy [kg]	0.02	0.91
Body mass at birth [kg]	0.17	0.44
Body mass increase during pregnancy [kg]	0.17	0.45
BMI before pregnancy [kg/m^2^]	0.04	0.88
BMI at birth [kg/m^2^]	0.15	0.52
BMI increase during pregnancy [kg/m^2^]	0.11	0.62
Newborn body mass [g]	−0.11	0.64
APGAR [0–10 points]	0.43	0.04

R_s_—Spearman rank correlation coefficient.

**Table 9:** Correlations between *KCNQ1* expression in the placenta and clinical parameters in the GDM group.

Parameters Correlated with Placental Expression of *KCNQ1*	Rs	*p*
Age [years]	0.001	1.00
Fasting glucose [mg/dl]	0.14	0.54
Daily insulin requirement [unit]	0.32	0.15
Body mass before pregnancy [kg]	0.17	0.45
Body mass at birth [kg]	−0.09	0.68
Body mass increase during pregnancy [kg]	−0.23	0.29
BMI before pregnancy [kg/m^2^]	0.14	0.53
BMI at birth [kg/m^2^]	0.01	0.96
BMI increase during pregnancy [kg/m^2^]	−0.25	0.27
Newborn body mass [g]	0.11	0.63
APGAR [0–10 points]	0.20	0.37
*KCNJ11* expression in the placenta	0.21	0.36

R_s_—Spearman rank correlation coefficient.

## Data Availability

Not applicable.
